# Antibiotic Use and Misuse in Dentistry in India—A Systematic Review

**DOI:** 10.3390/antibiotics10121459

**Published:** 2021-11-26

**Authors:** Aarthi Bhuvaraghan, Rebecca King, Harriet Larvin, Vishal R. Aggarwal

**Affiliations:** 1 Sree Balaji Dental College and Hospital, Bharat Institute of Higher Education and Research, Chennai 600100, India; dnabhu@leeds.ac.uk; 2 School of Dentistry, Faculty of Medicine & Health, University of Leeds, The Worsley Building, Clarendon Way, Leeds LS2 9LU, UK; dnhrl@leeds.ac.uk; 3 Nuffield Centre for International Health and Development, The Worsley Building, Clarendon Way, Leeds LS2 9LU, UK; r.king@leeds.ac.uk

**Keywords:** antimicrobial resistance, antibiotic resistance, AMR, antibiotic overuse, antibiotic misuse, inappropriate prescription, self-medication, dentistry, India

## Abstract

**Background**: Infections caused by antibiotic resistance pose a serious global health threat, undermining our ability to treat common infections and deliver complex medical procedures. Antibiotic misuse, particularly in low-–middle-income countries, is accelerating this problem. **Aim:** The aim of this systematic review was to investigate the use and misuse of antibiotics in dentistry in India. **Method**: We included studies carried out on Indian populations evaluating the prescription of prophylactic or therapeutic antibiotics by dental practitioners or other healthcare providers, along with antibiotic self-medication by the general population. The primary outcome measure was prescription rate/use of antibiotics for dental/oral problems. The secondary outcome measures included indications for antibiotic use in dentistry, their types and regimens, factors influencing practitioners’ prescription patterns and any differences based on prescriber and patient characteristics. Multiple databases were searched with no restrictions on language or publication date. The quality assessment of all included studies was carried out using the AXIS tool for cross-sectional studies and the Joanna Briggs Institute checklist for qualitative studies. **Results**: Of the 1377 studies identified, 50 were eligible for review, comprising 35 questionnaire surveys, 14 prescription audits and one qualitative study (semi-structured interviews). The overall quality of the included studies was found to be low to moderate. The proportion of antibiotic prescriptions amongst all prescriptions made was found to range from 27% to 88%, with most studies reporting antibiotics in over half of all prescriptions; studies also reported a high proportion of prescriptions with a fixed dose drug combination. Worryingly, combination doses not recommended by the WHO AWaRe classification were being used. The rate of antibiotic self-medication reported for dental problems varied from 5% to 35%. **Conclusions**: Our review identified the significant misuse of antibiotics for dental diseases, with inappropriate use therapeutically and prophylactically, the use of broad spectrum and combination antibiotics not recommended by WHO, and self-medication by the general population. There is an urgent need for targeted stewardship programmes in this arena.

## 1. Introduction

Infections caused by antimicrobial-resistant organisms kill at least 700,000 people every year, and could cause 10 million deaths by the year 2050, a loss of 100 trillion US dollars to the global economy, and a 2–3.5% reduction in the world’s GDP, if left unchecked [1]. They therefore constitute a serious global health threat that makes treating common infections as well as delivering complex medical procedures a challenge [2,3]. Although resistance is a normal evolutionary process for microorganisms, it is accelerated by the widespread use of antimicrobials [4]. Evidence suggests a clear link between the levels of antimicrobial use and the development of antimicrobial resistance [5,6].

Within the spectrum of antimicrobial resistance (AMR) comes antibiotic resistance, which refers to the ability of bacteria to survive in the presence of the antibiotic designed to kill them or stop them from multiplying [7]. This type of resistance is facilitated and accelerated by the use and misuse of antibiotics [8]. Any use, however small and appropriate, can facilitate resistance [9]. Infections such as tuberculosis, pneumonia and gonorrhoea and many food-borne diseases are becoming more difficult to treat because of resistant bacteria, resulting in increased mortality, high hospital costs and longer hospital stays [10]. This is compounded by the fact that no new class of antibiotics have been discovered since the late 1980s to counter these infections [11]. The problem is made worse when inappropriate usage occurs, for example when antibiotics can be bought over the counter without a prescription and/or where they are prescribed inappropriately by healthcare providers due to a lack of standard treatment guidelines.

Global antibiotic consumption rose by 40% between 2000 and 2010, and the BRICS countries (Brazil, Russia, India, China, South Africa) accounted for about three-quarters of this consumption [12]. Drivers of antibiotic resistance at a community level are a huge problem in India, where self-medication is commonplace and where antibiotics can be bought over the counter [13,14]. An estimated 56,524 neonates die in India of sepsis due to bacteria resistant to first-line antibiotics each year [15]. In fact, India, with one-sixth of the world’s population, was the largest consumer of antibiotics for human medicine in 2010 [12,16].

In developed countries, dental antibiotic prescriptions account for somewhere between 7% and 10% of the total antibiotics in healthcare [17,18,19,20,21]. However, a large proportion of antibiotic prescriptions have been found to be inappropriate [22,23,24,25,26,27]. Conditions such as toothache caused by pulpal or periapical inflammation do not need systemic antibiotics as they are localised conditions and are best managed by dental intervention such as tooth extraction or removal of the dental pulp [28]. Additionally, prophylactic use is no longer recommended routinely for dental procedures [29]. Inappropriate prescription puts the patient at risk of adverse events such as anaphylaxis and antibiotic-related colitis, in addition to the concerns around future drug-resistant infections in the population [28,30].

The use of antibiotics in dental practice is widespread in India [31]. Various studies evaluating dental practitioners’ knowledge and prescription patterns, conducted across India, indicated the inappropriate prescription of antibiotics [32,33,34,35]. India has a low dentist to population ratio [36], made worse by the unequal distribution of the available dental workforce [37]; therefore, healthcare often shifts to the hands of non-dental practitioners, such as informal healthcare providers (IHCP) [38], particularly in rural areas. Recent research on antibiotic prescriptions by IHCP showed that approximately 90% of all prescriptions for dental/oral problems contained antibiotics, a rate that was greater than that for any other health problem [38]. Worryingly, the rate of antibiotic use could be an underestimate, as it does not account for over-the-counter antibiotic use.

Considering the complexity of antibiotic consumption for dental problems in India, and a number of studies reporting inappropriate prescription, the full extent of this problem is unknown and the reasons for such prescription patterns are unclear and need further investigation.

While the data on antibiotic use in dentistry have been collected and are available in high-income countries, there data are lacking in low-–middle-income countries such as India [39]. The World Health Organisation (WHO), in its Global Action Plan on AMR, 2015, stressed the importance of collecting data on antibiotic use as one of the top priorities in tackling this growing problem [39].

This systematic review therefore aims to assess the use and misuse of antibiotics for dental problems in India.

The specific objectives are to determine:

1. The prevalence of prescribing antibiotics for dental problems;
2. Clinical (therapeutic/prophylactic) and non-clinical indications where antibiotics are prescribed in dentistry;
3. The types and regimen of antibiotics used;
4. The difference, if any, between rural and urban populations, adults and children, males and females, and socioeconomic classes;
5. Differences in antibiotic prescription based on provider characteristics;
6. The factors influencing practitioners’ prescription patterns; and
7. The reasons for self-medication with antibiotics and their sources.

## 2. Methods

### 2.1. Protocol Registration

The protocol for this systematic review was registered on PROSPERO (CRD42020165814) (https://www.crd.york.ac.uk/prospero, accessed on 16 March 2020).

### 2.2. Information Sources and Search Strategy

The following electronic databases were searched from their inception until 29th Feb 2020: Cochrane Database of Systematic Reviews, EMBASE, MEDLINE (Ovid), CINAHL, International Pharmaceutical Abstracts, Global Health, Web of Science and Google Scholar. The search was updated on 7 November 2021 to include any new studies. Supplementary Table S1 shows the search strategy for the above databases. Key search terms included antibiotic, antimicrobial, antibacterial, drug resistance, overuse, misuse, consumption, inappropriate prescription, self-medication, knowledge, stewardship, survey, dentistry, dentists, pharmacists, physicians, and health services.

Dissertation data (www.theses.com, accessed on 7 November 2021) and grey literature (www.opengrey.eu, accessed on 7 November 2021) were searched for on-going and unpublished studies. The reference lists of all eligible studies were checked for additional studies. Hand-searching of selected journals (Indian Journal of Dental Research, Journal of Indian Association of Public Health Dentistry) was performed in order to identify any missed studies. No language restriction was applied, and there was no restriction on the date of publication.

### 2.3. Inclusion Criteria

Studies carried out in India and on Indian populations and those that were available electronically were included. Studies that evaluated the prescription of prophylactic or therapeutic antibiotics by general dental practitioners, specialist dental practitioners (dentists with additional training and included in the Dental Council of India’s specialist register) or other healthcare providers for dental problems were considered eligible. Studies reporting on self-medication by the general population for dental problems were also included.

### 2.4. Exclusion Criteria

Exclusion criteria included:

- Case reports and case series;
- Studies involving dental students;
- Studies performed in vitro; and

Studies examining the use of antibacterial mouthwashes and other oral rinses, oral mucosal/gingival gels (antibiotic gels were included).

### 2.5. Research Question

Among adults and children with dental/oral health problems in India, what are the rates and indications for the use of antibiotics?

The following criteria (PICO—Population, Intervention/Exposure, Comparison, Outcome) were applied when considering studies for this review.

Population: Adults and children living in both urban and rural India, who:

- were seeking treatment from dental practitioners, dental specialists or other healthcare providers (including general medical practitioners, informal healthcare providers, etc.) for oral/dental problems; or
- had taken at least one course of antibiotics to help with dental/oral problems (irrespective of whether they completed the course or not), without consulting a dentist or other health practitioner (self-medication).

Intervention/Exposure: The review focused on exposure to antibiotics/consumption within the Indian population, both prescribed and self-medication.

Comparison: The study did not have a comparison arm.

Outcome measures: The primary outcome measures were the prescription rate of antibiotics for dental/oral problems in India and their indications. This also included the rate of over-the-counter antibiotic use (self-medication) for dental problems.

Secondary outcomes:

Where data were available, we evaluated:

1. Indications for dental antibiotic prescription: clinical (therapeutic/prophylactic) and non-clinical.
2. The types and regimens of antibiotics used.
3. The difference, if any, between rural and urban populations, adults and children, male and female, and socioeconomic classes.
4. The difference, if any, between prescriber (provider) characteristics (general dental practitioner/specialist dental practitioner/general medical practitioner/Informal healthcare provider; male/female; urban/rural).
5. The factors influencing practitioners’ prescription patterns, e.g., source of knowledge, such as monographs, textbooks and journals, colleagues, continuing professional development programmes, etc.
6. The sources of antibiotics, if self-prescribed, and the reasons for self-medication.

### 2.6. Study Screening and Selection

The screening and selection process was carried out in accordance with the Preferred Reporting Items in Systematic Reviews and Meta-Analyses [40]. After literature retrieval, studies were exported to EndNote (Clarivate Analytics) and deduplicated before review. Two authors (AB, HL) independently reviewed the titles and abstracts and excluded all irrelevant studies. Full texts were obtained for potentially eligible articles and those where a clear decision could not be made from the title and abstract alone. These articles were carefully examined for compliance with our inclusion and exclusion criteria. Any discrepancies in study inclusion were resolved after discussion with a third reviewer (VA).

### 2.7. Data Extraction and Synthesis

Data extraction for all included studies was performed independently and in duplicate by both the authors (AB, HL), and any discrepancies were resolved through discussion and consultation with a third reviewer (VA). The customised data extraction form was piloted and both authors (AB, HL) extracting the data participated in the piloting so that they were clear about the extraction process. The data extraction form included details on authors, year of publication, study setting, demographic details of prescriber and patients, rate of antibiotic use, details of antibiotics used and indications and details of self-medication. A sample data extraction form is attached in Supplementary Table S2. Changes and refinements to the form were carried out through discussion with all authors as part of the piloting process. Three groups of studies were identified in the literature that reported three key areas of antibiotic use for dental problems in India:

1. Studies exploring antibiotic prescription rates by dentists and other healthcare providers for dental problems;
2. Studies that investigated self-medication practices by the general population for dental/oral problems; and
3. Studies that explored indications, and the knowledge and practice of dentists in prescribing antibiotics for dental conditions.

### 2.8. Data Analysis

Data were descriptively analysed with mean percentages (standard deviations) for all quantitative outcomes. Forest plots were used where possible to display the effect sizes, and heterogeneity was determined using I2 analysis. The “meta” package in R version 4.0.0 was used to generate forest plots. Thematic analysis was performed for qualitative outcomes.

### 2.9. Rate of Antibiotic Use

To address this objective, we extracted data from prescription audits and hospital case record analysis of prescriptions and reported prescriptions containing antibiotics as a percentage of the total prescriptions made for dental/oral problems. The use of multiple antibiotics and/or fixed dose combinations was also identified and reported.

Where studies were carried out for other medical conditions, and included prescriptions for dental problems, we reported the proportion of these dental prescriptions compared to those issued for other medical conditions as a percentage.

To report the proportion of antibiotic self-medication, we extracted data from questionnaire studies involving the self-reported use of antibiotics for dental problems. These data were reported as percentages of the overall self-medicating population. Where the reasons for antibiotic self-medication were assessed for all health problems, the proportion of people self-medicating for dental problems was extracted.

### 2.10. Clinical and Non-Clinical Indications for Prescribing Antibiotics

We extracted data from questionnaire studies involving the antibiotic prescription patterns of dental practitioners for various dental/oral conditions. These data were tabulated as therapeutic clinical indications, where antibiotics were prescribed to treat dental disease (either alone or in combination with an operative intervention) and prophylactic clinical indications, to prevent infection either at the surgical site (e.g., minor dental surgeries) or at a distant site (e.g., prophylaxis for infective endocarditis).

Free-text responses related to clinical and non-clinical indications for prescribing (e.g., time constraints/patient pressures) were collated and reported quantitatively where possible. Where data were available, *t*-tests were used to examine significant differences between the antibiotic prescription pattern between groups (e.g., between dentists with and without postgraduate qualifications).

### 2.11. Types and Regimen of Antibiotics Used

We extracted data about the types of antibiotics used from knowledge-based questionnaire studies and prescriptions involving dental practitioners and informal healthcare providers and classified these according to the World Health Organisation AWaRe (Access, Watch, Reserve) Classification [41], and whether they were included in India’s National List of Essential Medicines 2015 [42]. The WHO AWaRe Database was developed in 2019 to enable the optimal use of antibiotics by countries and reduce antibiotic resistance. The Access group includes antibiotics with lower resistance potential and that are active against common pathogens. The Watch group antibiotics have a higher resistance potential and are key targets of stewardship programmes. The Reserve group are “last resort” antibiotics reserved for multi-drug-resistant organisms. In addition, the database also lists a number of fixed dose combinations of multiple broad-spectrum antibiotics whose use in not evidence-based and is therefore not recommended.

Where available, the antibiotic regimen (combinations used, frequency/dose) was extracted. Any differences in the pattern of antibiotic use (prescription/self-prescription) among various population groups (for example, rural/urban; adult/child; male/female; different socioeconomic strata) or among providers (dentists vs. specialist dentists vs. non-dental prescribers; male/female; rural/urban), where reported, were extracted.

To address the other objectives, data regarding factors influencing practitioners’ prescription pattern and their source of prescription knowledge were recorded.

From the studies involving self-medicating populations, the reasons for antibiotic self-medication for dental problems and the sources of antibiotics were extracted and tabulated.

### 2.12. Quality Assessment

The quality assessment of all included studies was carried out using the AXIS (Appraisal of Cross-sectional studies) tool [43]. The studies involving qualitative research were assessed using JBI (Joanna Briggs Institute) checklist [44].

The AXIS tool does not provide an overall numerical score to classify studies based on quality. However, in this review, we categorised the overall quality of included studies based on the following criteria for ease of understanding: a score of 17 or above was classified as high quality (low risk of bias), a score between 13 and 16 was classified as moderate quality (moderate risk of bias), and a score below 13 was considered low quality (high risk of bias).

## 3. Results

The search strategy identified 1226 studies, and after the removal of duplicates and title and abstract screening process, 96 studies were retrieved. A thorough citation search and website search resulted in the identification of a further 151 articles, of which 35 were included. Full-text analysis was performed for these 131 studies and 50 were considered as being eligible for inclusion in our review.

**Figure 1:**
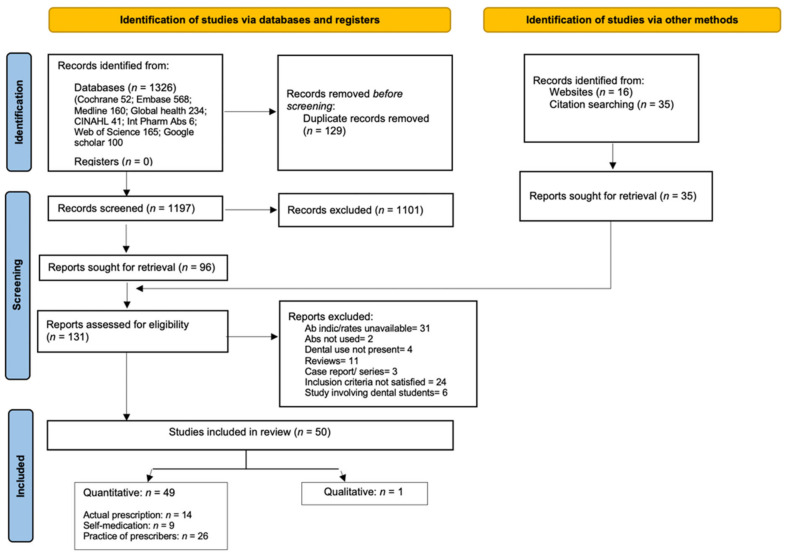
PRISMA flowchart.

Figure 1 shows the PRISMA flow chart for study screening and selection.

### 3.1. Study Characteristics

The characteristics of all included studies can be found in Table 1.

#### 3.1.1. Study Design

Of the fifty studies, 49 studies involved quantitative research and reported the number and percentages of antibiotic prescriptions and indications for use. One study involved qualitative research and assessed the perception of prescribers about drivers of antibiotic resistance in dentistry, through open-ended interviews.

Among the quantitative research, most were questionnaire surveys (*n* = 32) or prescription audits (*n* = 12). Two studies analysed hospital case records of patients, two were questionnaire-based interviews and one study involved simulated patients.

#### 3.1.2. Participants

Fourteen studies [38,45,46,47,48,49,50,51,52,53,54,55,56,57] assessed the prescribing rates for antibiotics by examining either prescription audits or dental outpatient hospital case records of patients. In 13 of these studies, the prescribers were dentists or dental specialists, while one study [38] examined the prescribing rate of informal healthcare providers for dental problems.

Nine studies [58,59,60,61,62,63,64,65,66] examined self-medication practices by the general population for dental/oral problems.

Twenty-six studies [32,33,34,35,67,68,69,70,71,72,73,74,75,76,77,78,79,80,81,82,83,84,85,86,87] explored the indications and reasons for antibiotic prescription/dispensing. Of these, 25 studies involved questionnaire surveys of dentists, and one study [38] involved antibiotic dispensing by pharmacists without a prescription to patients with dental complaints. Additionally, one study [88] was qualitative in nature, involving both dentists and pharmacists. This qualitative study adapted the grounded theory approach to understand the perceptions, beliefs and experiences of dentists and pharmacists.

**Table 1:** Characteristic table.

Actual Antibiotic Prescription										
Study	Location; Urban/Rural		Setting	Type of Population (Adult/Child) and Age Range	Male and Female %	Healthcare Provider/Prescriber	Number Eligible/Retrieved	Number of Prescriptions with Antibiotics	Outcome Evaluated	Outcome Evaluation Method
Bhattacharya, 2012	Bilaspur, Chattisgarh. Urban		3 primary care + 2 tertiary care hospitals	Adult (age ≥ 18 years)	n/r	Dentist	600/ 600	463	Drugs prescribed for toothache	Prescriptions
Chandy, 2016	Vellore, TN. Urban/Rural		Small hospitals, GP clinics, pharmacy shops	Adult/Child	n/r	GPs, pharmacists/dentists?	353/ 353	353 (all were Ab orescriptions	Pattern of antibiotic use in community—Ab use for various health problems (including dental) were assessed.	Prescriptions
Datta-Datta, 2015	Chennai, TN. Urban		Tertiary care teaching	Adult (age ≥ 18 years)	n/r	Oral Medicine specialist	300/ 300	Not available	Drug utilisation pattern of oral medicine department	Prescriptions
Deep Inder-Pawan Kumar, 2019	South Delhi. Urban		Tertiary care teaching	Adult/Child (>10 years)	68.5%, 31.5%	Dentist	783/ 1000	439	Drug utilisation pattern at dental outpatients	Prescriptions
Fayisa, 2019	Malappuram, Kerala. Rural		Tertiary care teaching	Adult/Child (5–63 years)	42.4%, 57.6%	Dentist	2802/ 2802	Not available	Drug utilisation and prescribing trends of antibiotics	Prescriptions
Jayanthi- Naidu, 2014	Mysore, Karnataka. Urban		Tertiary care teaching	Child (specific age range not reported)	n/r	Paediatric dentist/	600/ 600	160	Drug utilisation and cost analysis in paediatric outpatients	Prescriptions
Kaikade, 2016	Dhule, Maharashtra. Urban		Tertiary care teaching	Child (specific age range not reported)	n/r	Paediatric dentist	300/ 300	200	Antibiotic prescription pattern in paediatric dentistry outpatients	Prescriptions
Khare, 2019	Ujjain, MP. Rural		Primary care	Adult/Child (not reported)	n/r	Informal Healthcare Providers	1273/ 1273	1126	Practices and seasonal changes in antibiotic prescription for common illness	Prescriptions
Patel NN, 2014	Piparia, Vadodara, Gujarat. Rural		Tertiary care teaching	Adult/Child (not reported)	61.5%, 38.5%	Dentist	200/ 200	Not available	Utilisation pattern of antimicrobial agents	Patient interview and hospital case record
Patel PS, 2016	Vadodara, Gujarat. Urban		Tertiary care hospital	Adult/Child (not reported)	53.6%, 46.4%	Dentist	934/ 934	Not available	Drug utilisation pattern at dental outpatients department	Patient case records
Salman, 2009	Aligarh, UP. Urban		Tertiary care teaching	Adult/Child (not reported)	n/r	Dentist	Not reported	Not available	Drug prescribing pattern in the outpatients department	Prescriptions
Sharma M, 2014	Jaipur, Rajastan. Urban		Tertiary care teaching	Child (2–16 years)	n/r	Paediatric dentist	619/ 619	Not available	Drug prescribing pattern in paediatric dentistry outpatients	Prescriptions
Suhaib, 2017	Aligarh, UP. Urban		Tertiary care teaching	Adult/Child (11–70 years)	54%, 46%	Dentist	100/ 115	Not available	Antimicrobial prescription pattern in dental outpatients	Prescriptions
**Self-Medication**										
**Study**	**Location**	**Setting**	**Type of Population** (**Adult**/**Child**) **and Age Range**	**Male**, **Female %**	**Prescriber**	**Number Reported**/**Chosen** (**Response Rate**)	**Num using Abs**/**Total Self**-**Medicating**(**Antibiotic** **Self**-**Medication Rate**)	**Outcomes Evaluated**	**Outcome Evaluation Method**	Other **Outcomes Evaluated**
Dhaimade-Banga 2018	Tertiary care teaching hospital, Mumbai, Maharashtra.	Urban	Adults 25–70 years mean age 36.22	45.3%, 54.7%	Self	300/ 300	32/ 243	Prevalence of self-medication for dental problems	Questionnaire	Source of medication, reasons for self-medicating.
Giriraju, 2014	Tertiary care teaching hospital, Davangere, Karnataka.	Urban	Adults 18–65 years Mean age 38.8	75.6%, 24.4%	Self	410/ 410	22/ 312	Prevalence and perception about self-medication for oral health problems	Questionnaire	Source of medication, triggering factors, reasons for self-medicating, level of education and SES
Komalraj, 2015	Tertiary care teaching hospital, Bengaluru, Karnataka.	Urban	Adults ≥ 18 years Mean age 38.8 ± 12.76	61.7%, 38.3%	Self	175/ 175	12/ 175	Prevalence of self-medication for dental problems	Questionnaire	Source of medication, triggering factors, reasons for self-medicating, level of education and SES
Shamsudeen, 2018	Tertiary care teaching hospital, Chennai, TN.	Urban	Adults 18–65 years 36 ± 15.62	48.7%, 51.3%	Self	610/ 610	Not available	Prevalence, knowledge, practice of antibiotic self-medication	Interview- based on questionnaire	Source of medication, reasons for self-medicating.
Simon, 2015	Tertiary care teaching hospital, Manipal, Karnataka.	Rural	Adults 18–66 years 33.51 ± 12.98	34%, 66%	Self	400/ 400	10/ 120	Prevalence, pattern and awareness about self-medic for oral health problems	Interview based on questionnaire	Source of medication, triggers, reasons for self-medicating, level of education
Sultane, 2017	Tertiary care teaching hospital, Udaipur, Rajasthan.	Urban	Adults 18–65 years	56.8%, 43.2%	Self	220/ 220	78/154	Prevalence of self-medication for dental problems	Questionnaire	Source of medication, triggering factors, reasons for self-med, level of education
Gandhi	Tertiary care teaching hospital, Gujarat	Rural	Adults 21–60 years	51.3%, 48.7%	Self	230/ 230	Not available	Prevalence of self-medication for oral/dental problems	Questionnaire	Awareness about self-medication, and the risk factors among rural population
Rawlani	Tertiary care teaching hospital, Wardha	Rural	Adults 7–70 years	54.3%, 45.7%	Self	175/ 175	Not available	Prevalence of self-medication for dental problems	Questionnaire	Factors associated with self-medication for dental problems.
Mahmoud, M.A.	Hyderabad, Telangana state	Urban	Adults > 18 years	62.3%, 37.7%	Self	175/ 175	Not available	Prevalence of antibiotic self-medication in the community	Questionnaire	Reasons for antibiotic use, criteria for antibiotic selection and source of information, knowledge on impact of self-medication.
**Indications for Antibiotic Prescription**										
**Study**	**Location**		**Setting**	**Population Evaluated**	**Mean Age**/**Age Stratification**	**Male %**	**Number Reported**/**Chosen** (**Response Rate**)	**Outcome Evaluated**	**Type of Antibiotic**	**Outcome Evaluation Method**
Datta, 2014	Tertiary care teaching hospital, Mohali, Punjab.		Urban/Rural Primary and tertiary care; India-various	Dentists performing implant surgery	n/r	n/r	332/ 350	Antibiotics for routine implant placement	Prophylactic	Questionnaire
Garg, 2013	Tertiary care teaching hospital, Indore.		Urban/Rural Primary and tertiary care; India-various	Dental practitioners	31.58 ± 7.2 years	55.3%, 44.7%	552/ 1600	Pulp and periapical diseases	Therapeutic	Questionnaire
Goud, 2012	Tertiary care teaching hospital, Bhopal.		Urban/Rural Primary and tertiary care.	Dental practitioners	n/r	n/r	80/ 120	Various dental diseases and minor surgical procedures	Prophylactic + therapeutic	Questionnaire
Gowri, 2015	Tertiary care teaching hospital, Meerut, UP.		Urban Tertiary care	Interns, junior residents and specialist dentists.	n/r	n/r	120/ 120	Various dental diseases and minor surgical procedures	Prophylactic + therapeutic	Questionnaire
Jayadev, 2014	Tertiary care teaching hospital, Hyderabad.		Urban Primary and tertiary care.	Dentists	21–30 years 70.5%; 31–40 years 23.2%; 41–60 years 6.3%	51.4%, 48.6%	344/ 400	Pulp and periapical pathologies	Therapeutic + Prophylactic	Questionnaire
Karibasappa, 2014	Tertiary care teaching hospital, Dhule.		Urban Primary and tertiary care.	BDS and MDS qualified dentists	n/r	54%, 46%	82/ 82	Various oral conditions and routine dental treatment	Prophylactic + therapeutic	Questionnaire
Kaul, 2018	Tertiary care teaching hospital, Kolkata.		Urban Primary and tertiary care.	BDS and MDS qualified dentists	71% respondents were <30 years	62%, 38%	115/ 300	Various. Not clearly stated	Prophylactic + therapeutic	Questionnaire
Konde, 2017	Tertiary care teaching hospital, Bangalore.		Urban Primary and tertiary care.	Dental practitioners and paediatric dentists.	n/r	n/r	200/ 200	Various paediatric oral conditions	Prophylactic + therapeutic	Questionnaire
Kumar, 2013	Tertiary care teaching hospital, Secunderabad.		Urban Primary and tertiary care.	Dentists	28.6 ± 6.5 years (21–25 years 42.1%; 26–30 years 29.2%; 31–35 years 12.5%; 36–40 years 9.3%; 41+ years 6.9%	50%, 50%	216/ 246	Pulp and periapical pathologies	Therapeutic	Questionnaire
Peedikayil, 2012	Tertiary care teaching hospital, Kannur.		Urban/Rural Primary and tertiary care.	Dentists	36.7 ± 10.7 years (<25 years 19.35%; 26–40 years 47.58%; 41–55 years 29.03%; >55 years 4.03%	56.4%, 43.6%	248/ 300	Various dental infections and routine dental procedures	Prophylactic and therapeutic	Questionnaire
Saini, 2014	Tertiary care teaching hospital, Jaipur.		Urban/Rural Primary and tertiary care.	Dental practitioners	Mean age 41 years	n/r	500/ 525	Dental infection and routine dental procedures	Prophylactic and therapeutic	Questionnaire
Sam Prasad, 2017	Tertiary care teaching hospital, Chennai.		Urban. Primary care.	Dental practitioners	Mean 41.88 years. Age range 24–67 years.	57%, 43%	100/ 100	Unclear	Unclear	Questionnaire
Shafia, 2019	Tertiary care teaching hospital, Srinagar.		Urban. Primary and tertiary care.	GDPs and specialist dental practitioners	n/r	n/r	247/ 300	Various dental infections and routine dental procedures	Prophylactic and therapeutic	Questionnaire
Wasan, 2017	Tertiary care teaching hospital, New Delhi.		Urban Primary and tertiary care.	GDPs, specialist trainees and specialist practitioners	27.9 ± 7 years	41%, 59%	539/ 667	Various dental conditions	Prophylactic and therapeutic	Questionnaire
Gour, 2013	Tertiary care teaching hospital, Jaipur.		Urban Primary and tertiary care.	Dentists	n/r	56%, 44%	150/ 175	Various dental infections and prophylaxis	Prophylactic and therapeutic	Questionnaire
Harsh Vardhan, 2017	Tertiary care teaching hospital, Mallaram, Talangana.		Urban/Rural Primary and tertiary care.	Dentists and specialist dental practitioners	n/r	70%, 30%	450/ 700	Non-clinical reasons	N/a	Questionnaire
Nandkeoliar, 2016	Tertiary care teaching hospital, Imphal, Manipur.		Urban/Rural Primary and tertiary care.	Dentists	21–25 years 28%; 26–30 years 36%; 31–35 years 23%; 36–40 years 4%; >41 years 9%	n/r	100/ 122	Various acute and chronic dental conditions and routine dental procedures	Prophylactic and therapeutic	Questionnaire
Naveen, 2015	Tertiary care teaching hospital, Bangalore.		Urban Tertiary care	Dentists and specialist dentists	n/r	47%	202/ 245	Various dental infections and prophylaxis for medically compromised patients	Prophylactic and therapeutic	Questionnaire
Padda, 2016	Tertiary care teaching hospital, Ferozepur, Punjab.		Urban/Rural Primary care.	Dentists	n/r	60%	200/ 200	Antibiotics prescription for various clinical signs and dental conditions	Therapeutic	Questionnaire
Patait, 2015	Tertiary care teaching hospital, Sangamner, Maharashtra.		Urban Tertiary care	Dentists and specialist dentists.	n/r	n/r	41/ 42	Various dental conditions	Therapeutic	Questionnaire
Punj, 2018	Tertiary care teaching hospital, Mangalore.		Urban Primary care.	Dentists	n/r	57.8%	173/ Not known	Unclear	Prophylactic and therapeutic	Questionnaire
Puranik, 2018	Tertiary care teaching hospital, Bengaluru.		Urban Primary care.	Dentists	56% ≤ 35 years; 44% >35 years	54.3%	400/ 400	Various oral conditions and dental procedures	Prophylactic and therapeutic	Questionnaire
Srinivasan, 2017	Tertiary care teaching hospital, Vellore.		Urban Primary care.	Dentists	25–35 years 70%; ≥36 years 30%	54%	117/ 150	Various dental conditions and procedures and non-clinical reasons	Prophylactic and therapeutic	Questionnaire
Tripathi 2020	Tertiary care teaching hospital, Secunderabad,		Urban Primary and tertiary care	Dentists	25–34 years 77.9%, 35–44 y 16%, 45–54 years 3.1%, 55–64 y 1.5%, >65 y 1.5%	52.7%, 47.3%	363/568	Implant therapy and management of peri-implantitis	Therapeutic	Questionnaire (online)
Kaul. R. 2021	Tertiary care hospital, Manipur		Urban/ rural Primary and tertiary care	Dentists	20–30 years 63.4%, 31–40 y 30.8%, 41–50 years 4%, >51 years 1.8%	40.6%, 59.4%	276/400	Pain and infection control in children	Prophylactic	Questionnaire (online)
Savithra Prakash	Pharmacies		Urban, Primary care	Pharmacists	n/r	n/r	61/68	Dispensing for toothache/toothache with fever	Therapeutic	Simulated patients
Shoeb Ahmed	Hyderabad		Urban, variable	Dentists, pharmacists	23–60 years	60%	25/25	Perception about reasons for AMR	n/a	Interviews (qualitative research)

#### 3.1.3. Study Setting

The study setting was reported clearly as involving both urban and rural populations in one study [47], as rural in 6 studies (three prescription audits [38,48,52] and three self-medication audits [60,61,63]), and as urban in 16 (nine prescription audits [45,49,50,51,53,54,55,56,57], six self-medication audits [58,59,64,65,66,88], and one survey [87]). In the remaining 27 studies, the study setting was unclear.

### 3.2. Quality Assessment

The quality assessment of our included studies is summarised in Supplementary Table S3a,b.

All the studies except for the study involving a qualitative design [88] were assessed using the AXIS tool.

Of the 49 studies assessed using the AXIS tool, six studies were judged as having a low risk of bias [35,47,59,63,72,85], fifteen studies [34,38,48,53,57,58,60,62,64,70,78,83] were judged as having a moderate risk of bias and twenty-eight studies [33,45,46,47,49,50,51,52,54,55,56,61,67,68,69,71,73,74,75,76,77,79,80,81,82,84,89,90] were judged as having a high risk of bias. The qualitative study satisfied seven out of ten criteria of the JBI Critical appraisal tool.

Selection process, non-responders, response rate information and information on non-responders were the domains that frequently introduced bias. Additionally, the measurement tool used/piloted and the repeatability of the methods were the two domains frequently assessed as providing high levels of risk of bias in the included studies. However, in providing our specified outcome data, most studies reported this accurately and were deemed as having low/moderate risk regarding outcome reporting.

### 3.3. Primary Outcomes

#### Rate of Antibiotic Use for Dental/Oral Problems

- Rate of antibiotic prescriptions in clinical dental settings

**Figure 2:**
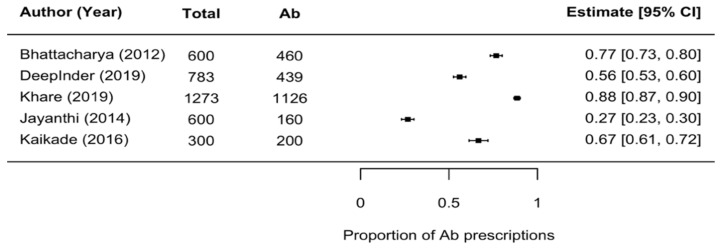
Proportion of antibiotic prescriptions within prescription audits. Key: antibiotics (Ab), confidence interval (CI).

The overall proportion of antibiotic prescriptions was reported by five prescription audits [38,45,49,50,51] involving 3556 prescriptions. Heterogeneity calculation that was carried out using *I*^2^ analysis showed a very high percentage (*I*^2^ = 99.53%). Figure 2 (Forest plot) shows that in four of these studies, over half of the prescriptions for oral/dental problems contained one or more antibiotics. In addition, three studies reported on the proportion of fixed drug dose combinations (FDCs) in all antibiotic prescriptions; of these, one study [50] involving a child population reported FDCs in 20% of antibiotic prescriptions, while the remaining two studies [48,52] involving rural adult populations reported 28% each. An FDC is a combination of two or more active pharmacological ingredients in a fixed ratio of doses [91].

b. Rate of over-the-counter antibiotic use (self-medication) for dental problems

**Figure 3:**
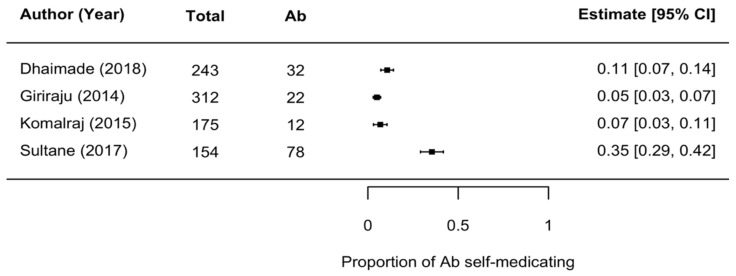
Proportion of self-medicating patients that reported usage of antibiotics. Key: antibiotics (Ab), confidence interval (CI).

Seven studies [58,59,60,61,63,64,65] explored the prevalence of self-medication practices for oral/dental problems in 1580 patients. Five of these studies [58,59,63,64,65] reported the proportion of patients self-medicating with antibiotics out of the total number of self-medicating patients in the study. Of these, one study involved a rural population [63] and reported antibiotic self-medication at a rate of 10%, while the remaining four involving urban settings (Figure 3) showed the spread of antibiotic self-medication to be between 5% and 35%. Similar to the antibiotic prescription rate, the heterogeneity calculations for the self-medication rate were also high (*I*^2^ = 98.80%). Additionally, 38.04% of self-medicating patients did not know what drug they were taking [60]. Only one study explored antibiotic self-medication in the community and found that 42.3% of the study population self-medicated with antibiotics for dental problems/pain, a proportion surpassed only by fever (76.8%) and cough and flu (70.8%) [66].

c. Rate of antibiotics prescribed in dentistry compared to other healthcare fields

Two studies [38,47] evaluated the percentage of antibiotic prescriptions for dental diseases compared to other medical conditions (Table 2).

**Table 2:** Rate of antibiotics prescribed in dentistry and other healthcare fields.

Author	Total Antibiotic Prescriptions in All Fields of (Human) Healthcare	Number of Prescriptions in Dentistry Alone	Proportion of Antibiotic Prescriptions Accounted for by Dentistry
Khare [38] (rural)	11,336	1126	9.93%
Chandy [47] (urban and rural)	10,800	353	3.3%

The study by Khare et al. [38] evaluated 11,336 antibiotic prescriptions of informal healthcare providers in a rural setting. The results show that 9.9% (1273 prescriptions) of all antibiotic prescriptions were for oral/dental problems (compared with 31.5% for fever (unspecified cause), 28.9% for upper respiratory tract infections, 11.2% for gastro-intestinal disorders and 7.5% for skin infections).

One study [47] that included both rural and urban populations identified 353 dental antibiotic encounters of a total of 10,800 antibiotic encounters in small hospitals, GP clinics and pharmacy shops. The dental reasons accounted for 3.3% of the overall antibiotic prescriptions/dispensations (compared to 21.2% for fever, 19.7% for upper respiratory tract infections, 11.5% for lower respiratory tract infections, 6.5% for gastrointestinal problems, 5.3% for skin and soft tissue problems, 4.8% for UTIs and 9% for wounds, 4% for cardiovascular reasons and 3.2% for surgery-related issues, among others) [47].

### 3.4. Secondary Outcomes

#### 3.4.1. Indications for Antibiotics

The various indications of antibiotic use identified from our studies are summarised in Table 3, Table 4, Table 5 and Table 6.

##### Therapeutic Indications for Antibiotic Prescription

Overall, twenty studies [32,33,34,35,56,68,69,70,71,72,73,74,75,76,77,83,85,86,87,89] assessed therapeutic and prophylactic indications for antibiotic use in dentistry.

Antibiotics were prescribed for therapeutic reasons in a number of acute and chronic dental and oral conditions/diseases. Periapical infection with spreading infection and systemic involvement (mean 85.7%, SD 12.46) [32,33,34,68,71,72,73,74,75,76,77,83,85] and a simple periapical abscess (mean 86.1%, SD 20.63) [32,69,72,73,74,77] were the most common conditions identified. A majority of dental practitioners prescribed antibiotics for acute conditions such as pulpal and periapical diseases. Acute pulpitis [32,34,73,74,75,77,83,85,89], irreversible pulpitis [32,33,68,71,73,74,77,89], acute apical periodontitis [33,68,71,73] and chronic apical periodontitis [33,68,71], necrotic pulp with sinus tract [32,33,68,71,73,77], pericoronitis [34,35,69,72,73,74,75,76,83], acute necrotising gingivitis [74,75,76], chronic periodontitis [34,73,74,75,89], periodontal abscess [34,69,74,75,83,85], acute [74,89] and chronic [75,83,89] gingivitis and dry socket [34,73,74,75,76,83,85] were the conditions that commonly received antibiotics. Antibiotics were also found to be prescribed for dental caries [35,72] and viral infections [35,70,73]. Other indications that were identified included sinusitis [83], trismus [56], tooth sensitivity [72], acute periodontitis [89], periodontal pocket [72] and halitosis [72] (Table 3).

**Table 3:** Therapeutic indications for antibiotic prescription.

Indication Identified	Proportion of Dentists Prescribing %	Mean Dentists’ Proportion Prescribing % (SD)
Acute pulpitis	30 [89], 13 [32], 71 [74], 43.6 [34], 76.5 [75], 49.1 [77], 63.8 [83], 60.8 [85]	50.98 (20.17)
Irreversible pulpitis	37.6 [68], 53 [89], 7.8 [71], 35 [32], 60.6 [33], 75 [74], 85.5 [77]	50.64 (26.34)
Pulpitis (non-specific)	72 [72], 54.8 [76], 23 [35]	50.26 (20.66)
Acute apical periodontitis	71.6 [68], 10 [71], 65.2 [33]	48.93 (27.65)
Chronic apical periodontitis	38.2 [68], 3.4 [71], 44.9 [33]	28.83 (18.19)
Apical periodontitis (non-specific)	87.8 [72], 85.5 [77], 39 [35]	70.7 (22.48)
Necrotic pulp/periapical abscess with sinus tract/discharge	46.9 [68], 15 [71], 57 [32], 69.4 [33], 55 [77]	48.66 (20.46)
Periapical/dentoalveolar abscess	98.8 [72], 50 [32], 95 [74], 98.7 [77], 88 [69]	86.1 (20.6)
Periapical abscess with extra oral swelling (includes space infection, cellulitis, spreading infection, systemic involvement)	90.2 [68], 56.4 [71], 97.6 [72], 70 [32], 92.1 [34], 93 [33], 91.6 [74], 82.5 [75], 76.2 [76], 98.5 [77], 88.8 [83], 91.9 [85]	85.7 (12.46)
Periodontal abscess	84 [69], 94 [74], 88.1 [34], 77 [75], 68.3 [83], 88.1 [85]	83.25 (8.42)
Pericoronitis	77 [69], 75.6 [72], 92 [74], 76.7 [34], 80 [75], 76.2 [76], 81.1 [83], 28.7 [35]	73.4 (18.83)
Soft tissue infections	90 [89]	90
Chronic periodontitis	33 [89], 65 [74], 47.5 [34], 51 [75]	48.63 (10.7)
Acute periodontitis	26 [89]	26
Acute gingivitis	23 [89], 74 [74]	48.5 (25.5)
Chronic gingivitis	3 [89], 50 [75], 28.2 [83]	27.07 (19.2)
Acute necrotising gingivitis	90 [74], 82 [75], 69 [76]	80.3 (8.65)
Dry socket	58 [74], 57.9 [34], 35 [75], 45.2 [76], 41.8 [83], 53.2 [85]	48.5 (9.36)
Dental caries	18.3 [72], 53 [35]	36.5 (17.35)
Viral infections	37.5 [70], 24.2 [35]	30.85 (6.6)
Other therapeutic indications identified: Sinusitis [83], trismus [56], tooth sensitivity [72], periodontal pocket [72], halitosis [72], peri-implantitis [87], and peri-implant mucositis [87].		

Patient symptoms were considered an important factor favouring the prescription of antibiotics [80,88].

##### Prophylactic Indications for Prescribing Antibiotics

Antibiotics were prescribed prophylactically to prevent post-operative infection in the operative site (primary prophylaxis) for routine dental procedures. Almost two-thirds of dentists prescribed antibiotics for routine dental extractions (mean 66.4%, SD 22.3) [34,35,69,72,73,74,77,81,83] and root canal treatment (mean 61.4%, SD 24.85) [34,35,71,72,76,77,81,82,83], respectively. Other procedures where antibiotics were commonly prescribed included the removal of impacted teeth (72.94, SD 27.63) [34,35,69,72,81,83,85], surgical extractions (52.1, SD 38.1) [35,72,73], routine implant placement (72.3, SD 27.4) [35,67,72,81], periodontal/flap surgery (71.25%, SD 27.17) [35,69,72,81], and periapical surgery (50.93, SD 32.42) [35,72,83]. Dentists also prescribed antibiotics for avulsed tooth replantation [32,83], tooth fractures [34,35,81], routine procedures such as scaling [74,81,83], and even asymptomatic impacted teeth [72] (Table 4).

**Table 4:** Prophylactic antibiotic prescription for dental procedures/conditions.

Prophylactic Indications for Prescribing Antibiotics		
Indication Identified	Proportion of Dentists Prescribing %	Mean Dentists’ Proportion Prescribing % (SD)
Tooth fracture/trauma	28.7 [34], 56.7 [35], 46.3 [81], 52.5 [86]	46.05 (10.7)
Scaling	2.5 [83], 42 [74], 18 [81]	20.8 (16.23)
Restoration	6 [86]	6
Periapical surgery	96.3 [72],22.5 [35], 34 [83]	50.93(32.42)
Extraction	67 [69], 72 [72], 91 [74], 84.7 [34], 72.6 [77], 76 [81], 13.6 [83], 54.5 [35], 39 [86], 26 [73]	59.64 (24.4)
Surgical extractions	90.2 [72], 14 [35]	52.1 (38.1)
Removal of impacted teeth	76.7 [69], 96.3 [72], 72.8 [34], 96 [81], 69.2 [83], 89.6 [85], 10 [35]	72.94 (27.63)
Periodontal/flap surgery	77 [69], 96.3 [72], 86 [81], 25.7 [35]	71.25 (31.37)
Minor oral surgeries	60 [70], 27.1 [77]	43.5 (16.45)
Soft tissue surgery	88 [71]	88
Routine Implants	85.5 [67], 92.7 [72], 86 [81], 25 [35]	72.3 (27.4)
Root canal treatment	84.1 [72], 78.7 [34], 71.4 [76], 20.9 [77], 60 [81], 76.6 [82], 88.8 [83], 27.7 [35], 44.8 [71]	61.4 (24.85)
Replantation of avulsed tooth	89 [32], 32.4 [83]	60.7 (28.3)
Other prophylactic indications identified: asymptomatic impacted tooth [72]; trauma to primary tooth [86], restoration of primary teeth [86], and extraction of primary teeth [86]		

Overall, approximately 57.1% of dentists admitted to prescribing prophylactic antibiotics routinely to prevent infection during dental procedures in healthy patients.

##### Antibiotic Prescription (Prophylaxis) in Medically Compromised Patients

Antibiotics were also commonly prescribed for patients with medical conditions such as type 1 diabetes (Mean 36.57%, SD 12.07) [35,73,83] and type 2 diabetes (Mean 72.47, SD 10.02) [81,82,85], blood dyscrasias (60.3, SD 33.68) [35,73,83] and pregnancy (37.07, SD 12.45) [74,81,82]. Other prophylactic indications identified were root canal treatment in medically compromised patients [34,69], hypertension [34,83], kidney transplant [34], liver failure [34], respiratory disorders [35,73], epilepsy [83], hyper and hypothyroidism [83], immunocompromised [74,83], carcinoma of large intestine [74], and infectious diseases [81].

Antibiotics were also prescribed to prevent infection in distant sites, such as in cardiac conditions.

We identified six such studies [32,34,35,69,74,83] which explored antibiotic prophylaxis in cardiac conditions. In general, a history of previous endocarditis [32,74,83], cardiac transplant [32], congenital and cyanotic cardiac diseases [32,73,74,83], mitral valve incompetence [74], prolapse with or without regurgitation [32], prosthetic heart valves [34,74], myocardial infarction [34,69], and the presence of a pacemaker [83] were the conditions where dentists reported prescribing prophylactic antibiotics. Additionally, rheumatoid arthritis [32] was also reported as a condition for which dentists would prescribe antibiotics prophylactically (Table 5).

**Table 5:** Prophylactic antibiotic prescription in medically compromised patients and cardiac conditions.

Antibiotic Prescription for Medically Compromised Patients		
Indication Identified	Proportion of Dentists Prescribing %	Mean Dentists’ Proportion Prescribing % (SD)
Medically compromised (unspecified)	3.3 [70]	3.3
Diabetes (Type 1)	45 [73], 19.5 [35], 45.2 [83]	36.57 (12.07)
Diabetes (Type 2)	78 [81], 81 [82], 58.4 [85]	72.47 (10.02)
Blood dyscrasias/bleeding disorders	76 [73], 13.5 [35], 91.4 [83]	60.3 (33.68)
Pregnancy	32 [81], 54.2 [82], 25 [74]	37.07 (12.45)
Other indications identified for medically compromised patients (prophylactic): RCT in medically compromised patients [34,69], hypertension [34,83], kidney transplant [34], liver failure [34], respiratory disorders [35,73], epilepsy [83], hyper- and hypothyroidism [83], immunocompromised [74,83], carcinoma of the large intestine [74], and infectious diseases [81]		

##### Non-Clinical Reasons for Prescribing Antibiotics

Nearly half of all dentists admitted to prescribing antibiotics for fear of losing patients (45.5, SD 7.5) [83,84,88], due to patient’s expectations (24.76, SD 21.71) [32,34,35,71,73,74,80,83,84,88], time constraints and workload (13.7, SD 14.71) [34,35,71,80], delaying or incomplete treatment (30.66, SD 18.57) [32,34,71,73,80], unsure diagnosis (32.68, SD 23.57) [34,71,74,80,82,83] and taking patient’s socioeconomic status into account (29.3, SD 19.4) [34,83] (Table 6). Dentists also reported considering patient’s oral hygiene and tobacco chewing habits before prescribing antibiotics [88]. Thematic analyses from the qualitative study [88] revealed key themes such as pressure from pharmaceutical companies, mutual commercial interests between pharmacy shop owners and dentists as reasons for prescribing antibiotics to dental patients.

**Table 6:** Non-clinical reasons for antibiotic prescription.

Non-Clinical Indication (Reasons) for Antibiotic Prescription		
Indication Identified	Proportion of Dentists Prescribing%	Mean Dentists’ Proportion Prescribing % (SD)
Patient expectation	5.6 [71], 4 [32], 35 [73], 57.32 [84], 45 [74], 8.4 [34], 5 [80], 7.5 [35], 55 [83], PNS [88]	24.76 (21.71)
Pressure of time and workload	7.8 [71], 5 [34], 3 [80], 39 [35]	13.7 (14.71)
Fear of loss of patient	38 [84], 53 [83], proportion not available [88]	45.5 (7.5)
Unsure diagnosis	36.1 [71], 14.5, 42 [74], 19.8 [34], 6 [80], 77.7 [83]	32.68 (23.57)
Delaying/incomplete treatment	34.79 [71], 9 [32], 51 [73], 49.5 [34], 9 [80]	30.66 (18.57)
Patient’s SES	9.9 [34], 48.7 [83]	29.3 (19.4)
Poor oral hygiene and patients’ habits (gutka chewing)	Proportion not available [88]	n/av
Market pressure from pharmaceutical companies and Mutual commercial interests.	5.6 [71], 4 [32], 35 [73], 57.32 [84], 45 [74], 8.4 [34], 5 [80], 7.5 [35], 55 [83], PNS [88]	24.76 (21.71)
Maintain dentist’s reputation	7.8 [71], 5 [34], 3 [80], 39 [35]	13.7 (14.71)

#### 3.4.2. Antibiotics Used

##### Types and Regimen of Antibiotics

Only a few studies provided the regimen of antibiotics used, and there was great variation in the type and regimen of antibiotics prescribed across studies (Supplementary Table S4).

Amoxicillin was found to be the most commonly used antibiotic for therapeutic and prophylactic indications, prescribed either alone or in combination with clavulanic acid.

In patients allergic to penicillin, erythromycin was the popular therapeutic choice, while clindamycin was the most popular prophylactic choice. Various generation cephalosporins, macrolides and quinolones were the second prophylactic choices [67,79,83], or were used in patients allergic to penicillin [32,33,34,68,71,74,75].

Doxycycline and metronidazole were preferred in periodontal management [34,69,75], although both these drugs were used for other dental indications as well. Metronidazole or a nitroimidazole antibiotic was often combined with other antibiotics such as amoxycillin or amoxicillin + clavulanic acid for anaerobic coverage [67,72,73].

**Figure 4:**
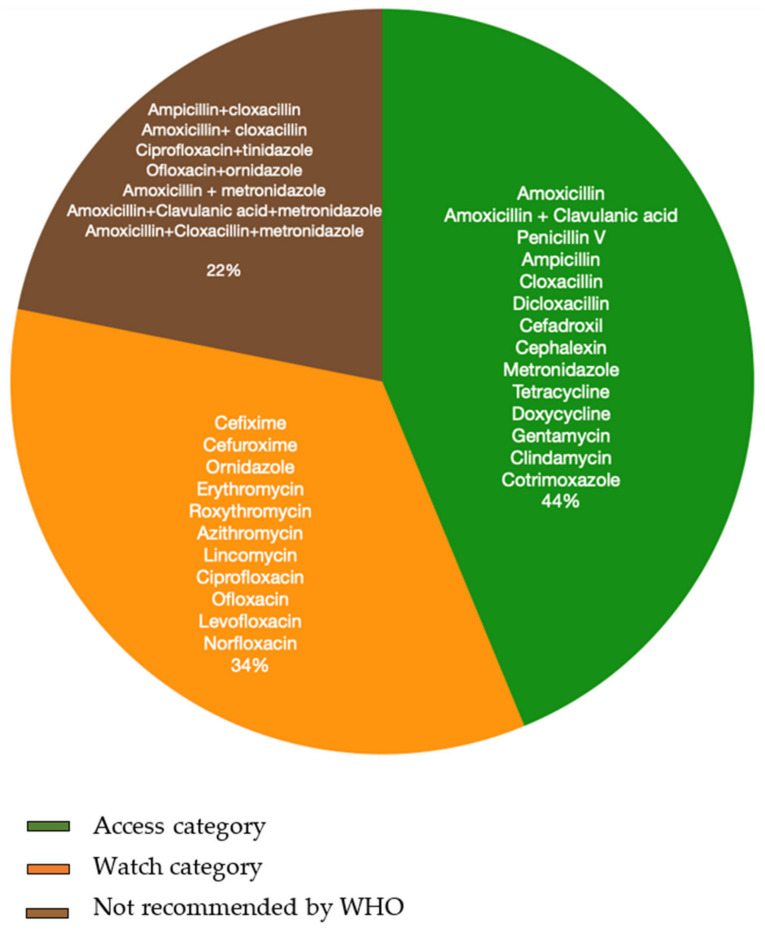
Type of antibiotics used in the Indian population based on the WHO AWaRe classification.

In total, 32 prescribing patterns were identified where antibiotics were prescribed either singly (*n* = 23) or in combination with other antibiotics or as a fixed dose combination of more than one drug/antibiotic (*n* = 9). Among the 23 individual antibiotics identified in our review, twelve are included under the WHO “Access” category and eleven under the “Watch” category (Figure 4) [41]. Thirteen of these 23 individual antibiotics are included in India’s National List of Essential Medicines (NLEM) 2015 [42]. Of the nine combinations/FDCs used, only two (amoxicillin/clavulanic acid and co-trimoxazole) are included in the ‘Access’ category as well as the NLEM of India. The WHO’s AWaRe [41] category does not recommend the use of the remaining seven antibiotic combinations in clinical practice (Figure 4).

Only four studies [38,51,52,79] reported the route of antibiotic administration. Oral administration was the most preferred choice for dentists, with a range of 86.5% to 100% prescribing antibiotics orally.

##### Combination Antibiotics and Fixed Dose Drug Combinations (FDC)

- Combinations Identified from Questionnaire Surveys

The proportion of dentists who reported prescribing combination antibiotics was identified from eleven questionnaire studies [32,67,68,70,71,72,74,76,77,81,85].

Amoxycillin/clavulanic acid is the most popular and favoured FDC, being the first choice of one-third of dentists from eleven studies [32,67,68,70,71,72,74,76,77,81,85]. Dentists often reported prescribing metronidazole in combination with amoxicillin [32,33,35,71] or amoxicillin/clavulanic acid (CA) [33,67,72,73], for anaerobic coverage.

While the use of ofloxacillin + ornidazole and ciprofloxacillin + tinidazole combinations has been identified in patients with and without a penicillin allergy, the former combination was also reported in child patients [73].

The mean percentage of practitioners prescribing combination antibiotics for dental/oral problems was 15.5% (SD 12.7) from 14 studies [32,33,67,68,70,71,72,73,74,76,77,80,81,85].

b. Combinations Identified from Prescription Audits (Actual Prescriptions)

The use of combination antibiotics was identified in five prescription audits. The proportion of such antibiotics varied between studies. The mean prescription rate for amoxicillin + clavulanic acid was found to be 15.9% (SD 16.04) [38,48,52,54], and that for ofloxacillin + ornidazole combination was 13.4% (SD 14.7) [45,52]. The study by Khare et al. reported the ampicillin + cloxacillin combination to be the most prescribed antibiotic for dental problems in a rural setting, albeit without giving any proportion [38].

#### 3.4.3. Antibiotic Use in Different Settings and Populations

- Difference in Antibiotic Prescription Rate between the Urban and Rural Population

Four studies [45,49,50,51] assessing 2283 prescriptions found the antibiotic prescription rate of dental practitioners in the urban population to vary between 27% and 77%.

Only one study reported the prescription rate in rural population where the prescribers were informal healthcare providers (IHCPs) [38]. This study assessed 1273 prescriptions, of which 88.45% contained at least one antibiotic (Figure 2).

b. Difference in Antibiotic Self-Medication Rates between Urban and Rural Population

The antibiotic self-medication rate was found to be between 5% and 35% in the urban setting among 862 self-medicating subjects from four studies (Figure 3) [58,59,64,65]. The rate in the rural setting was reported in one study only, and this was found to be 10% among 120 self-medicating people [63].

c. Difference in Antibiotic Prescription Rate between Adults and Children

The antibiotic prescription rate in adults from three studies [38,45,51] involving 2173 prescriptions ranged from 56% to 88%, whereas that in children was 27% and 67% from two studies [49,50] involving 900 prescriptions.

d. Difference in Prescription Rate Based on Prescriber Characteristics

Five studies compared antibiotic prescription rates for various clinical indications and dental procedures among dental practitioners with and without a postgraduate qualification [69,72,73,82,85].

Overall, 74.9% (SD 21.53) of BDS-qualified dentists and 52.1% (SD 25.6) of MDS-qualified dentists prescribed antibiotics for the indications identified (Table 7).

An independent sample *t*-test was used to compare the means between both the groups. The results show that MDS-qualified dentists prescribed statistically significantly fewer antibiotics compared to BDS-qualified dentists (Table 8).

#### 3.4.4. Factors Influencing Practitioners’ Prescription Pattern and/or Choice of Antibiotics

The various factors that influenced the prescription pattern of antibiotics were identified from four studies [35,80,83,84]. The most common factors were found to be the cost of the antibiotic and marketing factors, both of which were reported in three studies [31,48,74]. Surprisingly, only 20% of dentists took guidelines into consideration while prescribing antibiotics [68].

Dental practitioners’ knowledge about antibiotics came from various sources, such as university training, scientific societies [71,80], pharmacological companies [71], scientific literature [32], conferences and continuing dental education programmes [32], and textbooks and the Internet [32].

#### 3.4.5. Reasons for Self-Medication for Dental Problems

**Figure 5:**
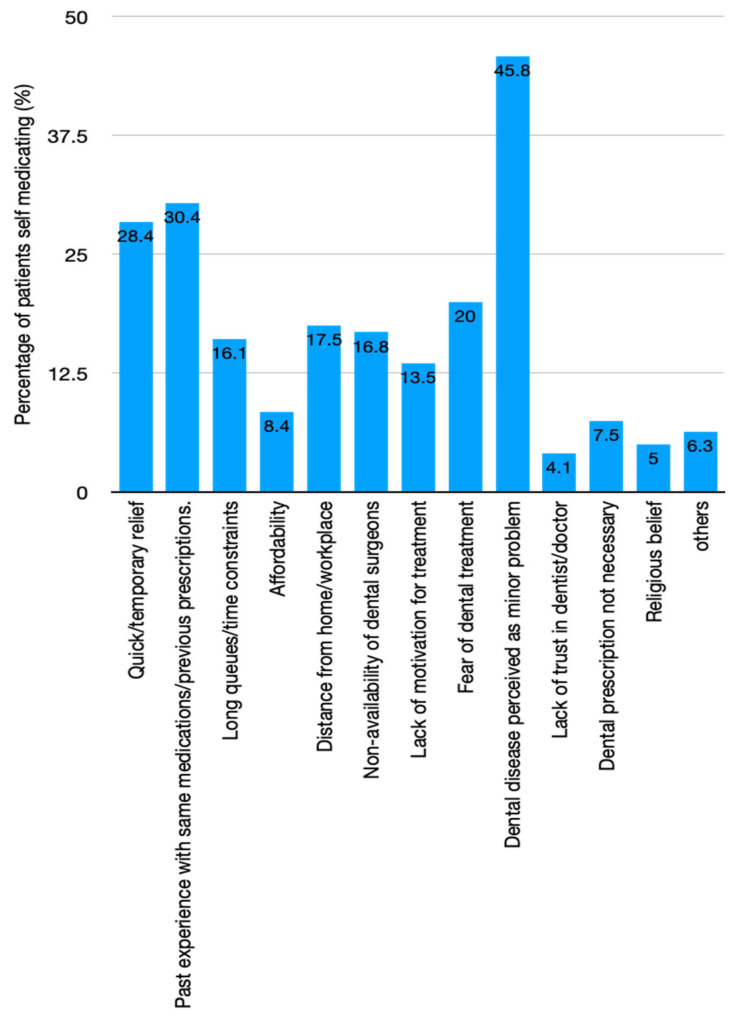
Reasons for self-medication by patients.

Dental patients report various reasons for resorting to self-medication for dental/oral problems, irrespective of whether they take antibiotics or other drugs or home/traditional remedies. The most important reason was considering their dental/oral complaints to be a minor problem [58,62,64,65]. The other common factors mentioned are fear of dental treatment [60,61], past experience and previous prescriptions [59,60,62,65], long queues in the dental clinical setting and time constraints [58,59,60,62,63,64,65], the distance of the dental practice [59,60,61] or non-availability of dental surgeons [60,61,63,64,65], among others. (Figure 5).

Reasons for Self-Medication with Antibiotics among Patients with Oral/Dental Problems

The qualitative study by Ahmed et al. [88] reported the reasons for using antibiotics (as self-medication) among patients with dental/oral problems.

The various reasons that emerged from this study were:

1. avoidance of the dentist;
2. easy accessibility to antibiotics without prescription and the ability to use these repeatedly as and when there is dental pain;
3. time constraints and cost of dental treatment;
4. immediate relief from dental pain,
5. mutual trust between the pharmacist and customers (dental patients), in the form of credits given by pharmacies to buy antibiotics, the ability of patients to return or replace antibiotics when they do not work.

## 4. Discussion

To the best of our knowledge, this is the first systematic review that explores the prevalence of the overuse of antibiotics for dental problems in India, a low—middle-income country which contains one-sixth of the world’s population. The key findings show high rates of antibiotic use, both through prescriptions and self-medication, inappropriate prescriptions, both therapeutically and prophylactically, and a readiness amongst the local population to use antibiotics for dental problems without consulting a dental professional. Worryingly, combination antibiotics which are not recommended by the WHO AWaRe classification were commonly used.

### 4.1. Antibiotic Prescription Rate

Our dental outpatient antibiotic prescription rate for adults ranged between 56% and 88%, which is much higher than the maximum accepted proportion of antibiotic prescriptions recommended by the WHO in any outpatient setting, which is 30% [92]. Additionally, the prescription rate for the child population was also high (66%) in Kaikade et al.’s study [50]. Our prescription rate is much higher when compared to 45.8% in England [93] and 57.4% in Germany [94]. This could be attributed to the stricter guidelines that are in place in these two countries. It is important to point out that the antimicrobial guidelines framed by the Indian Council of Medical Research, 2019, has no information on prescribing guidance in dentistry [95].

It is likely that heterogeneity estimations were high (*I*^2^ = 99.53%) due to the small number of studies within objectives. It is also possible that study factors such as location and populations may also contribute to significant heterogeneity; however, due to small sample sizes, we were not able to run subgroup meta-analyses. For this reason, meta-analysis was not performed.

### 4.2. Antibiotic Prescription in Dentistry versus Medicine

Only two of our included studies reported these data, and there was a wide variation between them. In both cases, the prescribers were not exclusively dentists. In the study by Khare et al. [38], dental patients accounted for 8% of all patients visiting IHCPs, but 10% of total antibiotic prescriptions. On the contrary, Chandy et al. [47] reported the antibiotic rate for dental problems as being 3.3% of all prescriptions. This difference could be attributed to the study location, as the former study reported on a rural population and the latter study reported on both rural and urban populations. Whilst these rates are comparable to the figures reported globally [17,18,19,20,21], the results must be taken with caution, as these data were from just two studies, and they also do not account for self-medication.

### 4.3. Self-Medication Rate

Although it is illegal to purchase antibiotics over the counter, we found in this review that self-medication with antibiotics was widely prevalent for dental problems in India. It could be argued that these figures could be much larger than they seem, as a significant proportion of patients who self-medicated did not know the names of the medicines they were taking [60].

Exploring the reasons for self-medication in India, Panda et al. reported that the perception of poor accessibility to healthcare, the chronic nature of disease and having a symptom count of more than two significantly increased the likelihood of using over the counter medication [96]. All of these factors are true with respect to dental disease. The results from our review are comparable with the antibiotic self-medication rates reported in other South East Asian Regions [97]. The rates in Pakistan [98] and Egypt [99] were 5.85% and 19.4%, respectively.

The high heterogeneity percentage (*I*^2^ = 98.80%) made these findings unsuitable for carrying out further meta-analysis.

### 4.4. Indications for Antibiotic Prescription

These data, synthesised from questionnaire studies, showed that dentists prescribed antibiotics for a number of acute and chronic dental conditions that clearly had no indications for antibiotic prescription and where local interventions such as draining the infection, removing the pulp or extracting the tooth would have sufficed. Inappropriate antibiotic prescription is not exclusive to India; it is a global problem and studies have found dentists’ poor adherence to antibiotic prescribing guidelines in developed countries [17,19,100,101,102] as well as developing countries [103].

Dentists reported prescribing prophylactic antibiotics to healthy patients for routine procedures such as scaling, simple extractions, minor surgical procedures and during root canal treatment. Unjustified use was reported in our review in certain medical conditions such as diabetes and hypertension, while in some conditions, routine prescription for dental procedures could be dangerous, as in the case of pregnancy and liver damage. Although the most common reason for prescribing prophylactic antibiotics was the prevention of infective endocarditis, our results show that dentists had poor knowledge of the guidelines. Furthermore, all our included studies were found to be published after 2007 AHA guidelines, but patients with prosthetic heart valves, mitral valve prolapse without regurgitation, congenital cyanotic heart diseases, myocardial infarction, pacemakers, and surprisingly rheumatoid arthritis were still prescribed prophylactic antibiotics for routine dental procedures when they should not have been. Again, this was similar to global trends [22,28].

The studies included in the review that assessed specific indications for prescription were obtained from self-reported questionnaire surveys. This type of study design introduces social desirability bias. The fact that respondents could have given answers that they believe as favourable could have led to our results being underestimates. Self-reporting, whether by providers or patients, always carries the risk of bias, however strong the study design is.

This review identified several non-clinical reasons including fear of losing patients, time constraints, training skills (unsure diagnosis, incomplete treatment), pressure from the patient on one side and market pressure from pharmaceutical companies on the other as leading dentists to prescribe antibiotics outside clinical indications. A recent umbrella review on the global population identified similar factors associated with antibiotic prescribing in acute dental conditions, including a “just in case” approach to prevent serious complications, peer influence, pressure from patients and impact of workload [23].

In this review, we found that a significant number of knowledge-based studies were conducted in tertiary care teaching institutions among teaching faculty with specialist qualifications. This could indicate a lack of knowledge among the trainers, which in turn calls into question the quality of the education passed on to the dental students regarding the appropriate use of antibiotics for dental diseases. Although some of our studies found that antibiotic prescription rates for specialist qualified dentists (MDS) were significantly lower compared to general dental practitioners (BDS), it must be emphasised that awareness regarding appropriate antibiotic prescription must begin in undergraduate training, and future interventions thus need to target undergraduate curricula to align them with current guidelines and antimicrobial stewardship in dentistry.

### 4.5. Types of Antibiotics

A total of 32 different prescribing patterns were identified. A number of them belonged to the WHO Watch category, which includes some critically important antibiotics that have a higher resistance potential [41].

The WHO discourages the use of fixed dose combinations of multiple broad-spectrum antibiotics, as it is not evidence-based. Seven out of nine antibiotic combinations identified in our study fell under this “not recommended” group. Moreover, over a quarter of antibiotic prescriptions in our review contained a fixed dose drug combination (FDC). The total antibiotic sales in India rose by 26% between 2007 and 2017, and FDCs contributed a major share to this, comprising a 38% increase, compared to a 20% increase in single drug formulations [104]. While FDCs help with treatment adherence in case of certain prevalent illnesses in India such as tuberculosis, malaria and HIV infection [105], their use for dental problems, especially in such high proportions, is not justified.

Although amoxicillin was the most popular therapeutic antibiotic in our review, similarly to previous studies on other populations [30,94], the use of broad-spectrum amoxicillin + clavulanic acid was also common among dentists in India. This is in contrast with the studies carried out in England and Germany, where amoxicillin+ clavulanic acid accounted only for 0.5% and 4.2%, respectively [30,94], of all antibiotics prescribed in dentistry. A recent systematic review on the global population identified both amoxicillin and amoxicillin + clavulanic acid to be popular therapeutic antibiotics in dentistry, similar to our review [106]. The increased use of broad-spectrum antibiotics could be attributed to their increased availability. The number of pharmaceutical companies manufacturing higher generation cephalosporins and amoxicillin + clavulanic acid was greater than the number of companies that manufacture amoxicillin [107]; in fact, only one company manufactured penicillin and benzathine penicillin [107].

While the consumption of broad-spectrum antibiotics, in general, is high in India [107], there has also been a rapid rise in the consumption of third-generation cephalosporins (WHO Watch category). Meanwhile, in contrast, the consumption of penicillins (WHO Access category) has remained stable [107]. Interestingly, the cost of some cephalosporins was found to be lower than that of Access group amoxicillin [107].

### 4.6. Providers

In all except two studies, the providers were found to be dentists. One study involved pharmacists dispensing antibiotics to standardised patients, and the other study involved informal healthcare providers in rural areas. The latter one had the highest reported antibiotic prescription rate of about 90%, calling into question their role in dental management. In developing countries such as India, the informal sector accounts for 51–96% of all providers and 9–90% of healthcare utilisation, especially for the poor population [108,109]. However, the quality of care has been found to be variable, and these providers were found to lack good knowledge, training or drug provision abilities, and their clinical practice often trailed behind with regard to knowledge [108].

In areas where dentists were not available or accessible, dental management often falls to non-dental providers such as IHCPs and general medical practitioners who are not trained to perform dental procedures, and therefore often tend to resort to antibiotics when patients present to them, especially with acute dental problems. As an untreated dental disease is chronic, patients are forced to take medication over and over again, until they are able to see a dental practitioner.

### 4.7. Role of Pharmacists

In developing countries, especially India, which is the second most populous country in the world, pharmacists are often approached by people for health advice, including dental advice. Shet et al. found that as high as 67% of private sector pharmacies in India dispensed antimicrobial drugs without prescriptions [110]. The reasons cited were convenience and easy access, cheaper cost, availability of credit and difficulty in getting an appointment with a dentist or physician [111]. It is understandable that in India, and other low middle-income countries, a significant proportion of the population may not be able to afford paying a qualified practitioner in addition to paying for medication. A questionnaire study involving pharmacists in India [111] showed that 22.4% of pharmacists would dispense antibiotics for toothache without a dentist’s prescription, as compared to 13% in Saudi Arabia [112]. The reason for the difference could be attributed to the economic differences and location of the study population.

This systematic review highlights various areas of antibiotic misuse in India namely inappropriate prescription, i.e., use of Watch category and combination antibiotics, the “just in case” approach of prescribing for dental conditions where antibiotics are not required, routine antibiotic use as prophylaxis in patients with medical conditions, and use in non-clinical situations; antibiotics dispensed by unqualified providers such as pharmacists and informal healthcare providers, and over-the-counter availability and use by the general population for dental problems.

## 5. Limitations

The main limitation of this review is the quality of the included studies. The overall quality of individual studies was low to moderate. All the included studies, except one, were cross sectional in nature and were prone to selection bias and confounding factors. Furthermore, as most studies involved questionnaires exploring self-reported knowledge and practice of prescribers, they are prone to social desirability bias, although the direction of this bias may be an underestimation of the extent of the existing problem, particularly in those who are aware of antimicrobial guidelines. That said, we used the studies only to provide descriptive data (percentages). We did not combine data from the studies to undertake meta-analysis, which would have been affected by the study quality, as well as the heterogeneity.

## 6. Future Research and Clinical Implications

This systematic review gives sufficient evidence that there is a substantial problem of overuse of antibiotics for dental problems in India, with antibiotics being prescribed inappropriately for clinical and non-clinical reasons; inappropriate, potent and combination antibiotics being prescribed; and antibiotics being obtained over the counter for dental problems by the general population. The review establishes the difference in antibiotic prescribing rates between general dentists and specialist qualified dentists, highlighting the need to emphasise the importance of optimal antibiotic prescribing in dental training.

A combination of factors including (i) provider issues such as a lack of knowledge, attitude, training, gaps in knowledge and practice, (ii) patient issues such as awareness and beliefs and (iii) policy issues such as guidelines, pharmacy regulations need to be addressed to bring about meaningful stewardship programmes. Further qualitative research is needed focussing on specific areas, for example, providers, the teaching faculty, pharmacists, policy makers or patients, to understand their knowledge, beliefs and barriers to antibiotic use and misuse. There is also a need to develop anti-microbial stewardship programmes to enable providers to appropriately prescribe, and patients to avoid self-medication.

## 7. Conclusions

Antibiotic misuse in dentistry is a serious global threat, with inappropriate use by both dental and non-dental healthcare professionals in India. The use of combination antibiotics and self-medication for dental problems was also alarming. Considering the serious problem of antibiotic resistance, there is an urgent need to address this overuse of antibiotics for dental/oral problems in India, using interventions that are targeted at both healthcare professionals and the general public to enable a nationwide change in this area.

## Figures and Tables

**Table 7:** Difference in prescription rates based on prescriber characteristics (qualification).

Study ID	Treatment	Prescription Rate among General Dentist (BDS)	Prescription Rate among Specialists (MDS)
Goud [69]	RCT	50	40
	Surgical removal of impacted teeth	76	80
Karibasappa [72]	Periodontal pocket	74.5	48.1
	Tooth fracture	54.5	29.6
	Pulpitis	89	37
	Apical periodontitis	96.4	70.4
	Periapical abscess	98.2	85.2
Konde [73]	Reversible pulpitis	28	2
	Irreversible pulpitis	84	36
	Apical periodontitis	96	71
	Simple extraction	45	7
	Periapical abscess	94	78
	Dry socket	96	45
Shafia [82]	RCT	83.6	69.6
Wasan [85]	Acute pulpitis	65.6	50.4
	Dry socket	54.9	50
	Periodontal abscess	88.6	87.1

**Table 8:** Independent sample *t*-test for prescriber characteristics.

Groups	Number of Clinical Indications	Mean	Standard Deviation	Mean Difference	95% Confidence Interval of Mean Difference		*p* Value
					Upper	Lower	
Dentist	17	74.9	21.53	22.81	−39.36	6.7	0.009
Specialist	17	52.1	25.6				

## Data Availability

Data will be made available upon reasonable request to the corresponding author.

## Supplementary Materials

The following are available online at https://www.mdpi.com/article/10.3390/antibiotics10121459/s1, Table S1: Search strategy used to identify relevant papers; Table S2: Data Extraction Forms; Table S3a,b: Quality assessment of included studies; Table S4: Types and Regimen of Antibiotics identified.
