# Supplementary material for: Antibiotic Use and Misuse in Dentistry in India—A Systematic Review
Source: Antibiotics (Basel). 2021 Nov 26;10(12):1459. doi: 10.3390/antibiotics10121459 (PMC8698453; doi:10.3390/antibiotics10121459)
Supplement: Supplementary file 1 [file antibiotics-10-01459-s001.zip › antibiotics-1428920-supplementary.pdf]

SUPPLEMENTARY DATA

Table S1. Search strategy used to identify relevant papers.

a) Databases – MEDLINE (Ovid), EMBASE, Global Health, International Pharmaceutical Abstracts.

(antibiotic* or antimicrobial* or antibacterial*).ti,ab.
2. exp Anti-Bacterial Agents/
3. exp Drug Resistance, Microbial/
4. 1 or 2 or 3
5. (prescri* or overprescri* or underprescri* or self-prescri* or "use" or usage or overuse or misuse or abuse or consumption or consume or rate or audit or survey or questionnaire* or pattern or trend* or stewardship or knowledge or appropriate or inappropriate or prophyla* or therap*).ti,ab.
6. exp Prescriptions/ or exp Drug Prescriptions/
7. exp "Surveys and Questionnaires"/
8. exp health knowledge, attitudes, practice/ or exp "treatment adherence and compliance"/
9. exp Antibiotic Prophylaxis/
10. exp inappropriate prescribing/ or exp self medication/
11. exp community health services/ or exp emergency medical services/ or exp rural health services/
12. exp allied health personnel/ or exp personnel, hospital/ or exp pharmacists/ or exp physicians/
13. 5 or 6 or 7 or 8 or 9 or 10 or 11 or 12
14. (dental or dentist* or odontogenic or tooth or teeth or orofacial or dentoalveolar or implant or "p*ediatric dental" or endodonti* or periodont* or pulp* or periapical or "root canal" or extraction*).ti,ab.
15. (oral adj2 (surg* or infection* or disease* or problem* or healthcare or health or infection* or abscess*)).ti,ab.
16. exp Dentists/
17. exp Dentistry/
18. 14 or 15 or 16 or 17
19. India*.ti,ab.
20. exp India/
20. 19 or 20
22. 4 and 13 and 18 and 21

b) Database - Cochrane.

#1 (antibiotic* or antimicrobial* or antibacterial*):ti,ab,kw 43984
#2 MeSH descriptor: [Drug Resistance, Microbial] explode all trees 2343
#3 #1 OR #2 45302
#4 (prescri* or overprescri* or underprescri* or self-prescri* or "use" or usage or overuse or misuse or abuse or consumption or consume or rate audit or survey or questionnaire* or pattern or trend* or stewardship or knowledge or appropriate or inappropriate or prophyla* or therap*):ti,ab,kw 1109004
#5 MeSH descriptor: [Prescriptions] explode all trees 1076
#6 MeSH descriptor: [Inappropriate Prescribing] explode all trees 166
#7 MeSH descriptor: [Drug Misuse] explode all trees 247
#8 MeSH descriptor: [Health Knowledge, Attitudes, Practice] explode all trees 6205
#9 MeSH descriptor: [Surveys and Questionnaires] explode all trees 57141
#10 MeSH descriptor: [Self Medication] explode all trees 87
#11 MeSH descriptor: [Practice Patterns, Physicians'] explode all trees 1280
#12 MeSH descriptor: [Antibiotic Prophylaxis] explode all trees 1316
#13 MeSH descriptor: [Allied Health Personnel] explode all trees 1255
#14 MeSH descriptor: [Community Health Services] explode all trees 14636
#15 MeSH descriptor: [Emergency Medical Services] explode all trees 4194
#16 MeSH descriptor: [Rural Health Services] explode all trees 355
#17 #4 OR #5 OR #6 OR #7 OR #8 OR #9 OR #10 OR #11 OR #12 OR #13 OR #14 OR #15 OR #16 1117247

#18 (dental or dentist* or odontogenic or tooth or teeth or orofacial or dentoalveolar or implant or "p*ediatric dental" or endodonti* or periodont* or pulp* or periapical or "root canal" or extraction*):ti,ab,kw 64467
#19 (oral adj2 (surg* or infection* or disease* or problem* or healthcare or health or infection* or abscess*)):ti,ab,kw 0
#20 MeSH descriptor: [Dentists] explode all trees 102
#21 MeSH descriptor: [Dentistry] explode all trees 18363
#22 #18 OR #19 OR #20 OR #21 65985
#23 India*:ti,ab,kw 13112
#24 MeSH descriptor: [India] explode all trees 2295
#25 #23 OR #24 13112
#26 #3 AND #17 AND #22 3573
#27 #25 AND #26 52

c) Database- CINAHL.

S1 (MH "Antibiotics+") OR (MH "Drug Resistance, Microbial+")
S2 TI ((antibiotic* or antimicrobial* or antibacterial*)) OR AB ((antibiotic* or antimicrobial* or antibacterial*))
S3 S1OR S2
S4 (MH "Inappropriate Prescribing") OR (MH "Prescriptions, Drug+") OR (MH "Self Medication")
S5 (MH "Surveys+") OR (MH "Questionnaires+")
S6 (MH "Attitude to Health+") OR (MH "Patient Compliance+") OR (MH "Dentist Attitudes")
S7 (MH "Personnel, Health Facility+") OR (MH "Allied Health Personnel+") OR (MH "Physicians+") OR (MH "Pharmacists") OR (MH "Health Personnel, Unlicensed")
S8 (MH "Emergency Medical Services+") OR (MH "Community Health Services+") OR (MH "Rural Health Services")
S9 (MH "Antibiotic Prophylaxis")
S10 TI (prescri* or overprescri* or underprescri* or self-prescri* or "use" or usage or overuse or misuse or abuse or consumption or consume or rate or audit or survey or questionnaire* or pattern or trend* or stewardship or knowledge or appropriate or inappropriate or prophyla* or therap*) OR AB (prescri* or overprescri* or underprescri* or self-prescri* or "use" or usage or overuse or misuse or abuse or consumption or consume or rate or audit or survey or questionnaire* or pattern or trend* or stewardship or knowledge or appropriate or inappropriate or prophyla* or therap*)
S11 S4ORS5ORS6ORS7ORS8ORS9OR S10
S12 (MH "Dentists+")
S13 (MH "Dentistry+")
S14 TI (dental or dentist* or odontogenic or tooth or teeth or orofacial or dentoalveolar or implant or "p*ediatric dental" or endodonti* or periodont* or pulp* or periapical or "root canal" or extraction*) OR AB (dental or dentist* or odontogenic or tooth or teeth or orofacial or dentoalveolar or implant or "p*ediatric dental" or endodonti* or periodont* or pulp* or periapical or "root canal" or extraction*)
S15 TI (oral n2 (surg* or infection* or disease* or problem* or healthcare or health or infection* or abscess*)) OR AB (oral n2 (surg* or infection* or disease* or problem* or healthcare or health or infection* or abscess*))
S16 S12ORS13ORS14ORS15
S17 (MH "India")
S18 TI India* OR AB India*
S19 S17 OR S18
S20 S3 AND S11 AND S16 AND S19

d) Database- Web of Science.

antibiotic* or antimicrobial* or antibacterial* (Topic) and
prescri* or overprescri* or underprescri* or self-prescri* or "use" or usage or overuse or misuse or abuse or consumption or consume or rate or audit or survey or questionnaire* or pattern or trend* or stewardship or knowledge or appropriate or inappropriate or prophyla* or therap* (Topic) and

dental or dentist* or odontogenic or tooth or teeth or orofacial or dentoalveolar or implant or "p*ediatric dental" or endodonti* or periodont* or pulp* or periapical or "root canal" or extraction* (Topic) and India* (Topic)

Table S2. DATA EXTRACTION FORM - ANTIBIOTIC USE.

Study Name:

Author: **Year:**

Journal/type of publication:
Funding source: **Not reported ✔**
Aim:
Study Design: **Cross-sectional**
Other (please specify)
Study Setting:
Primary care
Tertiary care hospital **Urban/Rural**
Tertiary care teaching hospital
Health Centre
Research Institute
Other: ____________________
Study Population:
Approach used: Questionnaire/prescription audit/ Other
Study period and duration:
Translation to local language/language of convenience done (if applicable): Yes/No – English/Hindi/Marathi/Kannada
Inclusion criteria:
Exclusion criteria:
EVALUATING ACTUAL PRESCRIBING PATTERN OF ANTIBIOTICS
Number recruited:
Number reported:
Child/Adult
Male/Female/both
Urban/Rural
U/UM/LM/UL/L
Demographic details (if any)
Age range =
Mean age =
Male: Female =
Statistical analysis:
OUTCOME
Rate of antibiotic prescription among all study participants =
Rate of prescriptions with antibiotics =
Rate of antibiotics prescribed among all prescription drugs (as often more than one antibiotic can be included in a prescription) =
Antibiotic combinations (more than 1 antibiotic) =
Indications for Antibiotic prescription

Author & Year	Indications for Antibiotic prescription	
	Clinical indications (therapeutic(T)/Prophylactic (P)	Non-clinical reasons (if any)

	Name	T/P	n	%	Name	n	%	Type and regimen (if available)	Rate

EVALUATING SELF-MEDICATION
Number recruited:
Number reported:

Child/Adult
Male/Female/both
Urban/Rural
U/UM/LM/UL/L
Male: Female
Age range
Mean age:

Statistical analysis:
OUTCOMES :
Prevalence of self-med for oral/dental problems: n= (%)
Most common age group (if available):
Prevalence of antibiotic use among all self-medications:
Reasons for self-medication:
Sources of drugs
Effect of self-medication
Side effects and their rate (if any mentioned)
Knowledge about Ab use and AMR:
Emerging themes:
EVALUATING KNOWLEDGE/ PRACTICE OF PRESCRIBERS
Population assessed:
Number recruited:
Number reported:
Response rate:
Male: Female =

Demographic Characteristics of Study Population								
Qualifications (Dental)					Gender			
UG students	Vocational Trainees/ interns	Graduates and PG trainees	Specialist qualification	Work experience	M N=	M %	F N =	F% =

OUTCOME

Condition/conditions assessed	T/P*	N =	%	Antibiotics used (if mentioned separately for acute /chronic conditions; penicillin allergic pts)	N=	%

* T= Therapeutic; P= Prophylactic.

Statistical analysis:
Antibiotic of choice = ------------ (n= ; % =)
Dose and duration (regimen)=
Antibiotic of choice for penicillin allergy patients = ------------ (n= ; % =)
Dose and duration =
Adverse reactions to antibiotics reported = (%)
% of subjects confident in prescribing antibiotics=
Based on questionnaire, %age showing good knowledge in prescribing drugs =
M = (%); F = (%)
Sources of prescription information:
Notes and themes:
ARTICLE INCLUDED FOR ANALYSIS: YES/NO

Table S3. a: Quality Assessment of Included studies using AXIS tool.

Study	Outcome	1. Clear aims	2. Study design	3. Sample size	4. Defined target Popln defined	5. Representative sample	6. Selection process	7. Non-responders	8.Measurementsappropriate	9. Correctly measured/piloted	10. Statistical significance	11. Methods Repeatable	12. Basic data	13. Response rate concerns	14. Non-responders' info	15. Results consistent	16. All analysis described	17. Discus' n/concl' n justified	18. Limitations	19. Conflicts	20. Ethics/ consent	Overall score
Bhattacharya 2012	Ab prescription	Y	Y	Y/nc	Y	Y	N	N	Y	N	N	N	N	D N	N	N	N	N	N	N	Y	8
Borole 2013	Ab prescription	N	Y	N/nc	N	N	N	N	N	N	N	N	N	D N	N	N	N	N	N	N	N	3
Chandy 2016	Ab prescription	Y	Y	Y/nc	Y	Y	Y	N/a	Y	Y	Y	Y	Y	n/a	n/a	Y	Y	Y	Y	N	Y	20
Datta 2015	Ab prescription	Y	Y	Y/nc	Y	Y	N	N	Y	Y	N	Y	N	DN	N	N	Y	N	N	N	Y	11
DeepInder 2019	Ab prescription	Y	Y	Y/ nc	Y	Y	d/ n	d/n	Y	Y	N	N	N	DN	DN	N	N	N	Y	N	Y	10
Fayisa 2019	Ab prescription	Y	Y	Y/nc	Y	Y	Y	N	Y	Y	N	Y	Y	DN	N	N	N	N	Y	N	Y	13
Jayanthi 2014	Ab prescription	Y	Y	Y	Y	Y	Y	D N	Y	Y	Y	Y	N	D N	DN	N	N	N	N	N	Y	12
Kaikade 2016	Ab prescription	Y	Y	Y/nc	Y	Y	N	Ndn	Y	Y	N	N	N	DN	D N	N	N	N	N	N	Y	9
Khare 2019	Ab prescription	Y	Y	Y?	Y	Y	N	D N	Y	Y	Y	Y	Y	DN	D N	Y	Y	Y	Y	N	Y	16
Patel NN 2014	Ab prescription	Y	Y	Y/nc	Y	Y	N	N	Y	Y	N	N	Y	DN	N	N	Y	N	N	N	Y	11
Patel PS 2016	Ab prescription	Y	Y	Y	Y	Y	Y	NA	Y	Y	N	N	N	N	NA	Y	N	N	Y	N	Y	15
Salman 2009	Ab prescription	Y	Y	N	Y	N	N	N	N	N	N	N	N	n/r	N	N	N	N	N	N	N	4
Sharma 2014	Ab prescription	Y	Y	Y	Y	Y	N	N	Y	Y	N	N	N	DN	N	N	Y	N	N	N	Y	10
Suhaib 2017	Ab prescription	Y	Y	N	Y	Y	N	Ndn	Y	Y	Y	Y	Y	N	Y	Y	Y	N	N	N	N	14
Dhaimade 2018	Self-medication	Y	Y	Y	Y	Y	N	n/ a	Y	Y	Y	Y	Y	N	n/a	N	Y	Y	N	N	Y	17
Giriraju 2014	Self-medication	Y	Y	Y	Y	Y	N	n/a	Y	N	N	Y	Y	N	n/ a	N	Y	Y	N	N	Y	15
KomalRaj 2015	Self-medication	Y	Y	Y	Y	Y	N	n/ a	Y	N	N	N	Y	N	n/a	N	Y	Y	N	N	Y	14
Shamsudeen 2018	Self-medication	Y	Y	Y	Y	Y	Y	d/n	Y	Y	Y	Y/ dn	N	D/ n	d/n	N	Y	N	N	N	Y	13
Simon 2015	Self-medication	Y	Y	Y	Y	Y	N	n/ a	Y	Y	Y	Y	Y	N	n/a	N	Y	Y	Y	N	Y	18
Sultane 2017	Self-medication	Y	Y	Y	Y	Y	N	n/ a	Y	Y	N	Y	Y	N	n/ a	N	Y	N	N	N	Y	15
Gandhi 2016	Self-medication	Y	Y	Y	Y	Y	Y	D N	Y	Y	N	Y	Y	N/dn	N/ dn	Y	Y	N	N	N	Y	14
Rawlani 2015	Self-medication	Y	Y	Y	Y	Y	N	d/n	Y	N	Y	Y	Y	N/dn	N/dn	N	N	N	Y	N	Y	12
Mahmoud MA 2019	Self-medication							N/dn						DN	DN							12
Datta 2014	Prescriber Knowledge/practice	Y	Y	N	Y	Y	N	N	Y	N	N	N	N	N	N	N	Y	Y	N	N	Y	10

Garg 2013	Prescriber Knowledge/practice	Y	Y	N	Y	N	Y	N	Y	N	Y	N	N	Y	N	N	Y	Y	N	N	Y	10
Goud 2012	Prescriber Knowledge/practice	Y	Y	Y	Y	Y	N	N	Y	Y	Y	N	N	Y	N	N	Y	Y	N	N	Y	12
Gour 2013	Prescriber Knowledge/practice	Y	Y	N	Y	Y	N	N	Y	Y	N	N	N	N	N	N	Y	N	N	N	Y	10
Gowri 2015	Prescriber Knowledge/practice	Y	Y	N/ dn	Y	Y	Y	n/a	Y	Y	Y	N	N	N	n/a	Y	N	N	N	N	Y	14
Harsh Vardhan 2017	Prescriber Knowledge/practice	Y	Y	N	Y	N	N	N	N	N	Y	Y	N	Y	N	N	Y	Y	N	N	Y	9
Jayadev 2014	Prescriber Knowledge/practice	Y	Y	N	Y	Y	N	N	Y	N	N	N	Y	N	N	N	Y	Y	N	N	Y	11
Karibasappa 2014	Prescriber Knowledge/practice	Y	Y	Y	Y	N	Y	n/a	Y	Y	Y	Y	Y	N	n/a	Y	Y	N	N	N	Y	17
Kaul 2018	Prescriber Knowledge/practice	Y	Y	N	Y	N	N	N	Y	Y	Y	N	Y	Y	N	N	Y	N	Y	N	Y	11
Konde 2017	Prescriber Knowledge/practice	Y	Y	N	Y	Y	N	DN	Y	Y	Y	N	N	DN	DN	N	N	N	N	N	Y	9
Kumar 2013	Prescriber Knowledge/practice	Y	Y	N	Y	N	Y	N	Y	N	Y	N	Y	N	N	N	Y	N	N	N	Y	11
Nandkeoliar 2016	Prescriber Knowledge/practice	Y	Y	Y	Y	N	Y	N	Y	N	N	N	N	N	N	N	Y	Y	Y	N	Y	12
Naveen 2015	Prescriber Knowledge/practice	Y	Y	Y	Y	Y	N	N	Y	Y	N	N	N	N	N	Y	Y	Y	Y	N	Y	14
Padda 2016	Prescriber Knowledge/practice	Y	Y	N	Y	Y	N	N/A	Y	Y	N	N	N	N	N/A	N	Y	N	N	N	Y	12
Patait 2015	Prescriber Knowledge/practice	Y	Y	N	Y	N	N	N	Y	N	N	N	N	N	N	Y	N	N	N	N	Y	8
Peedikayil 2012	Prescriber Knowledge/practice	Y	Y	N	Y	N	N	N	Y	N	N	Y	Y	N	N	N	Y	Y	Y	N	Y	12
Punj 2018	Prescriber Knowledge/practice	Y	Y	N	Y	Y	N	N/d	Y	N	N	Y	Y	D/N	N/ dn	N	N	N	N	N	Y	9
Puranik 2018	Prescriber Knowledge/practice	Y	Y	Y	Y	Y	N	N/A	Y	Y	Y	Y	Y	N	N/A	Y	Y	N	Y	N	Y	18
Saini 2014	Prescriber Knowledge/practice	Y	Y	N	Y	Y	N	N	N	N	N	N	N	N	N	N	N	N	N	N	Y	7
Sam Prasad 2017	Prescriber Knowledge/practice	Y	Y	N	Y	Y	N	N/A	N	N	N	N	Y	N	N/A	N	N	N	N	N	Y	10
Shafia 2019	Prescriber Knowledge/practice	Y	Y	Y	Y	Y	N	N	Y	N	Y	N	N	N	N	N	N	N	N	N	Y	10
Srinivasan 2017	Prescriber Knowledge/practice	Y	Y	N	Y	N	Y	N	Y	Y	Y	Y	Y	N/ dn	N	N	Y	Y	Y	N	Y	14
Wasan 2017	Prescriber Knowledge/practice	Y	Y	Y	Y	Y	N	N	Y	Y	Y	Y	Y	N	Y	N	Y	Y	Y	N	Y	17
Savithra Prakash	Prescriber (pharmacist) Knowledge/practice	Y	Y	N/d n	Y	Y	Y	N	Y	d/ n	N	Y/ dn	N	N	N	N/ dn	Y	Y/dn	Y	N	Y	13
Tripati R 2020	Prescriber Knowledge/practice			N/d n				N						Y	N	Y	Y	Y	Y	N		14
Kaul R 2021	Prescriber Knowledge/practice													N					Y	N	Y	16

Table S3. b: Quality assessment for qualitative study (Ahmed S) using JBI Critical Appraisal tool.

Assessment	Yes	No	Unclear	Not Applicable
1. Is there congruity between the stated philosophical perspective and the research methodology?		✓		
2. Is there congruity between the research methodology and the research question or objectives?	✓			

3. Is there congruity between the research methodology and the methods used to collect data?	✓			
4. Is there congruity between the research methodology and the representation and analysis of data?	✓			
5. Is there congruity between the research methodology and the interpretation of results?			✓	
6. Is there a statement locating the researcher culturally or theoretically?	✓			
7. Is the influence of the researcher on the research, and vice- versa, addressed?		✓		
8. Are participants, and their voices, adequately represented?	✓			
9. Is the research ethical according to current criteria or, for recent studies, and is there evidence of ethical approval by an appropriate body?	✓			
10. Do the conclusions drawn in the research report flow from the analysis, or interpretation, of the data?	✓			

Table S4. Types and regimen of antibiotics identified.

Scheme	**Type of Antibiotic**	**Regimen Used**	**WHO AWaRe Category**	**Drug Included in WHO National Essential Drugs List, India**
1	Amoxycillin [31–33,37,44,45,51–53,55,56,65–75,77,79,81,83,85,86]	250mg bd or tds<br>500mg bd or tds	Access	Y
2	Amoxicillin + Clavulanic acid [31,32,37,51–53,65–70,72,74,75,79,80,83,86]	375 mg tds<br>500mg bd<br>625 mg bd/ tds [69]	Access	Y
3	Penicillin V [73]	NS	Access	N
4	Ampicillin [52,68]	NS	Access	Y
5	Cloxacillin [68]	NS	Access	Y
6	Dicloxacillin [68]	NS	Access	N
7	Amoxicillin+ cloxacillin [32,54,55,65,67–69,75]	250mg + 250mg tds<br>250mg + 500mg tds	N/R by the WHO	n/a
8	Ampicillin with cloxacillin [37,52,68,83]	NS	N/R by the WHO	n/a
9	Amoxicillin + metronidazole [31,32,67,69,78]	250mg + 400 mg [32,69]<br>500 mg + 400mg [32]	N/R by the WHO	n/a
10	Amoxicillin+<br>Clavulanic acid 625mg + metronidazole 400mg [32,70,71]	Bd/ tds	N/R by the WHO	n/a
11	Amoxicillin+<br>Cloxacillin 500mg + metronidazole 400mg [32]	bd	N/R by the WHO	n/a
12	Cefadroxil [32,65,72]	500 mg bd	Access	Y
13	Cephalexin [32,65,68–72,77]	500mg bd	Access	N
14	Cefixime [32,53,56,65,68,72,83]	200 mg od/ bd	Watch	Y
15	Cefuroxime [65]	NS	Watch	N
16	Cephalosporins Unspecified [74,78]	NS	variable	variable
17	Metronidazole [32,33,37,44,45,51–53,55,65–68,70,73–75,83]	400 mg tds	Access	Y
18	Ornidazole [32,56]	500mg Bd	Watch	N

19	Tetracycline [32,37,45,68,85,86]	NS	Access	N
20	Doxycycline [32,33,45,51,56,65,67–69,73,74,78,85]	100mg Od/ bd	Access	Y
21	Erythromycin [31–33,65,66,68,69,72,73,81]	500 mg bd<br>500 mg od/ tds	Watch	Y
22	Roxythromycin [53,68]	NS	Watch	N
23	Azithromycin [31,32,52,53,65,66,68,72,83]	500 mg od/ tds	Watch	Y
24	Lincomycin [65]	NS	Watch	N
25	Gentamycin [67,74]	NS	Access	Y
26	Clindamycin [31,32,52,65,66,68,71,73,77,79,81]	300 mg bd	Access	Y
27	Ciprofloxacin [32,33,52,53,56,65,67–69,72,74,75,83]	200 mg bd<br>500 mg bd/ tds[69]	Watch	Y
28	Ofloxacin [32,56,65,68,74,79,83]	200 mg bd<br>400 mg bd	Watch	N
29	Levofloxacin [32,53]	500mg od	Watch	Y (listed under antitubercular medicines)
30	Norfloxacin [65]	NS	Watch	N
31	Cotrimoxazole (trimethoprim + sulfamethoxazole) [68]	NS	Access	Y
32	Ciprofloxacin+ tinidazole [66,68,72,74,83]	500mg + 300mg bd<br>500+ 600 mgbd	N/R by the WHO	n/a
33	Ofloxacin+ ornidazole [31,44,51,66–72,79,83]	200mg + 500mg bd/ tds	N/R by the WHO	n/a

Y- Yes; N- No; N/R Not Recommended; n/a not applicable (as combination antibiotics); Access: This group includes antibiotics that have activity against a wide range of commonly encountered susceptible pathogens while also showing lower resistance potential than antibiotics in the other groups; Watch: This group includes antibiotic classes that have higher resistance potential and includes most of the highest priority agents among the Critically Important Antimicrobials for Human Medicine (ref, critically important meds, 6th revision 2018), and/or antibiotics that are at relatively high risk of selection of bacterial resistance. These medicines should be prioritized as key targets of stewardship programs and monitoring. .
